# Trade-Offs of *Escherichia coli* Adaptation to an Intracellular Lifestyle in Macrophages

**DOI:** 10.1371/journal.pone.0146123

**Published:** 2016-01-11

**Authors:** M. Azevedo, A. Sousa, J. Moura de Sousa, J. A. Thompson, J. T. Proença, I. Gordo

**Affiliations:** Instituto Gulbenkian de Ciência, Rua da Quinta Grande n°6, Oeiras, Portugal; Leiden University, NETHERLANDS

## Abstract

The bacterium *Escherichia coli* exhibits remarkable genomic and phenotypic variation, with some pathogenic strains having evolved to survive and even replicate in the harsh intra-macrophage environment. The rate and effects of mutations that can cause pathoadaptation are key determinants of the pace at which *E*. *coli* can colonize such niches and become pathogenic. We used experimental evolution to determine the speed and evolutionary paths undertaken by a commensal strain of *E*. *coli* when adapting to intracellular life. We estimated the acquisition of pathoadaptive mutations at a rate of 10^−6^ per genome per generation, resulting in the fixation of more virulent strains in less than a hundred generations. Whole genome sequencing of independently evolved clones showed that the main targets of intracellular adaptation involved loss of function mutations in genes implicated in the assembly of the lipopolysaccharide core, iron metabolism and di- and tri-peptide transport, namely *rfaI*, *fhuA* and *tppB*, respectively. We found a substantial amount of antagonistic pleiotropy in evolved populations, as well as metabolic trade-offs, commonly found in intracellular bacteria with reduced genome sizes. Overall, the low levels of clonal interference detected indicate that the first steps of the transition of a commensal *E*. *coli* into intracellular pathogens are dominated by a few pathoadaptive mutations with very strong effects.

## Introduction

Bacterial populations have an enormous potential to adapt to their environments. This is inferred from studies of molecular evolution and variation that find signatures of selection in many genes [1,2]. The remarkable pace of bacterial adaptation can also be directly demonstrated in the laboratory by following evolution in real time, over many generations, in controlled environments with specific selection pressures [3–5]. Many studies of microbial evolution in real time involve studying adaptation to simple abiotic environments consisting of single or multiple sugars [6,7], characterizing compensation to the costs of deleterious mutations, such as antibiotic resistance genes in drug free environments [8,9], or studying adaptation in spatially structured environments [10–12]. Complex environments, in which multiple, more natural, selective pressures are present, have received far less attention [13]. The vast majority of these experiments demonstrate the acquisition of adaptive mutations at high rates, with swift genetic and phenotypic changes. One way to quantify these evolutionary parameters is by following the dynamics of neutral markers in evolving clonal populations, where rapid and large allele frequency changes indicate the occurrence of a high rate adaptive mutations with strong selective effects [14–16].

Rapid adaptation is also detected in pathogen populations colonizing humans during infection [17]. In these natural environments, where bacteria are likely to encounter many different types of cells, key antagonistic interactions are imposed by the host innate immune system. Overcoming these interactions is often part of the transition from commensalism to pathogenesis [18,19]. Different strains of *E*. *coli* can be either commensals or versatile pathogens, and even switch between the two, and there is increasing evidence that some pathogenic strains evolved from commensal *E*. *coli* [20,21]. Several natural *E*. *coli* pathovars have been studied, some of which use common mechanisms to increase their virulence. Many of such virulence traits are encoded in pathogenicity islands (blocks of genes found in a pathogen but not in related nonpathogenic strains [22,23]), plasmids or prophages, highlighting the importance of successful horizontal gene transfer in pathogen adaptation to new niches. In addition to gene acquisition, gene loss can also contribute to the emergence and diversity of existing *E*. *coli* pathovars [24], as well as other genome modifications which may lead to increased bacterial pathogenesis in the absence of horizontal transfer. These are usually called pathoadaptative mutations [25]. For instance, the knockout of *hemB*, an hemin biosynthetic gene, in *Staphylococcus aureus*, which leads to increased ability to persist intracellularly, constitutes a pathoadaptive mutation and mutations in *hemL* of *E*. *coli*, encoding glutamate-1-semialdehyde aminotransferase, can also confer pathogenic properties [Ramiro, Costa and Gordo, submitted]. Another common pathoadaptive mutation is the loss of the gene *mucA*, which in *Pseudomonas aeruginosa* increases its ability to evade phagocytosis and resist to pulmonary clearance [26]. In another remarkable example, Koli and colleagues [27] showed that two genetic changes in commensal *E*. *coli* K-12 were sufficient to reprogram its cellular transcription and render it invasive in eukaryotic cells, both *in vivo* and *ex vivo*. Macrophages (MΦs), one of the major cell types of the innate immune system, are a typical intracellular niche for certain *E*. *coli* pathovars, including *Shigella*, enteroinvasive *E*. *coli* (EIEC) and adherent-invasive *E*. *coli* (AIEC). The former, for instance, is commonly found in patients of Crohn’s disease, can adhere to intestinal epithelial cells and invade and survive in epithelial cells and macrophages [28]. Characterization of these pathoadaptive mutations is therefore important to understand the emergence of bacterial pathogenesis. We have previously studied the short-term adaptation of *E*. *coli* to recurrent encounters with macrophages and found that mucoid clones, which carry IS1 insertions into the regulatory region of *yrfF* and that overproduce colanic acid, repeatedly evolved [29].

Here, we use experimental evolution to study *E*. *coli* adaptation to the intra-macrophage environment and to dissect the possible initial adaptive steps for a bacterium to adopt such a lifestyle. We used an established two-marker system to study bacterial adaptation *in vitro* and to determine the rate and fitness effects of pathoadaptive mutations. We then characterized phenotypically the bacteria that evolved and used whole genome sequencing to determine the most likely pathoadaptive evolutionary paths for the first steps in the transition into an intracellular environment.

## Materials and Methods

### Ethics statement

All experiments involving animals were approved by the Institutional Ethics Committee at the Instituto Gulbenkian de Ciência (project nr. A009/2010 with approval date 2010/10/15), following the Portuguese legislation (PORT 1005/92) which complies with the European Directive 86/609/EEC of the European Council. Endpoints to euthanize the animals were defined prior to the experiment. The specific signs used to make the decision of euthanizing the animals were: weight drop of 20% and/or body temperature decrease below 28°C (for two consecutive days). Despite the frequent monitoring of the animals’ health (at least two times a day), the aforementioned signals were not observed in any of the animals and, therefore, there was no need to perform euthanasia.

### Strains and media

The murine macrophage cell line RAW 264.7 (Sigma-Aldrich) was maintained in RPMI 1640-GlutaMAX I (Gibco) supplemented with 1 mM Sodium Pyruvate (Invitrogen), 10 mM HEPES (Invitrogen), 100 U/ml penicillin/streptomycin (Gibco), 50 μM 2-mercaptoethanol solution (Gibco), 50 μg/ml Gentamicin solution (Sigma) and 10% heat-inactivated FBS (standard RPMI complete medium). Culture conditions were at 37°C in a 5% CO_2_ atmosphere.

All bacterial cultures were grown in the same conditions as the macrophage line but using only 100μg/mL of streptomycin (RPMI-Strep medium) instead of the three antibiotics present in RPMI complete medium. The same medium was used for the infection assays of MΦs with bacteria. The *Escherichia coli* strains used were MC4100-YFP and MC4100-CFP (MC4100, galK::CFP/YFP, Amp^R^ Strep^R^), which express constitutively either the yellow (yfp) or the cyan (cfp) alleles of GFP integrated at the *galK* locus in MC4100 (*E*. *coli* Genetic Stock Center #6152) [15]. Unlike certain pathogenic *E*. *coli* strains, our commensal strain is a derivative of K12 which is not able to replicate within macrophages [27].

### Evolution Experiment

The evolution experiment was started from two single colonies of either MC4100-YFP or MC4100-CFP grown in RPMI-Strep in the same conditions as the cell line. The two bacterial cultures were mixed in equal proportion (5x10^3^ colony forming units (cfu) each) and used to infect the activated MΦs, in 20 replicates.

Before the infection MΦs were centrifuged at 201 g for 5 min and re-suspended in RPMI-Strep. After this step ~ 10^5^ cells per well were used to seed a 24-well microtiter plate and incubated over-night at 37°C with 5% CO_2_. Subsequently, activation was done by adding 2 μg/ml of CpG-ODN 1826 (5´TCCATGACGTTCCTGACGTT 3´—Sigma) [30] and incubating at 37°C with 5% CO_2_ for 24h. Following activation, cells were washed and infected with 10^4^ bacteria mix (multiplicity of infection (MOI) = 1 cfu: 10 MΦs). After infection we centrifuged the plates at 201 g for 5min (to increase the contact between MΦs and bacteria) and then incubated at 37°C with 5% CO_2_ for 24h [31]. Next we discarded the extracellular bacteria, washed the MΦs with RPMI-Strep two times and added 100μg/mL of Gentamicin solution or 1h at 37°C with 5% CO_2_ [32]. Gentamicin penetrates poorly the macrophages and therefore whereas intracellular bacteria are protected from the bactericidal action of the antibiotic the extracellular are killed [33]. After washing out the gentamicin with PBS 1X, cells were lysed using a 0.1% Triton-X–PBS solution for 15 minutes [34]. Intracellular bacteria were collected, washed with PBS 1X and counted by flow cytometry using LSR Fortessa cytometer (BD Biosciences). From approximately 10^6^ intracellular bacteria collected, we pooled 10^4^ and infected a new batch of activated MΦs, in the same manner as described previously. This procedure was repeated for 26 days, a period after which fixation of one of the fluorescent markers could be observed for most of the replicate experiments, an indication of adaptation. This propagation protocol allows ~ 7 generations per day, calculated by Log_2_ (Nf/Ni), where Nf is the number of intracellular bacteria 24h post-infection, and Ni is the bacterial inoculum used to infect the macrophages [29].

### Fitness measurements

Fitness increases of the evolved populations were estimated by competitive fitness assays in the presence or in the absence of MΦs. A sample of 30 clones carrying the fluorescence marker which achieved the highest frequency in a given population was competed against the ancestral strain labeled with a different marker. These samples of clones were assumed to be representative of the population. The competition assays for each evolved population were done in triplicate in the same conditions as the evolution experiment, for two passages—48h. The neutrality of the fluorescent marker was tested by competition of the two ancestral strains (9 replicates).

Relative fitness, expressed as a selection coefficient, was estimated by calculating the slope of the natural logarithm of the ratio of evolved over ancestral bacteria per generation of ancestral bacteria [35].

### Whole genome re-sequencing and mutation prediction

#### Ancestral genome

The sequence reads were mapped to the reference strain *Escherichia coli* K12 MG1655 BW2952 (reference NC_012759.1). The extra mutations carried by the two ancestors in relation to the reference are described in [29].

#### Clone analysis

In the last time point of the evolution experiment, we isolated a clone from each evolved population carrying the fluorescent marker with higher frequency. In the populations where both markers reached similar frequencies at the last time point, one clone from each marker subpopulation was isolated. Each of these clones was then grown in 10mL of RPMI at 37°C. DNA isolation from these cultures was subsequently obtained according to [36].

The DNA library construction and sequencing was carried out by the in-house genomics facility. Each sample was paired-end sequenced using an Illumina MiSeq Benchtop Sequencer. Standard procedures produced data sets of Illumina paired-end 250bp read pairs. Genome sequencing data have been deposited in the NCBI Read Archive http://www.ncbi.nlm.nih.gov/sra (accession no. SRP066892). The mean coverage per sample was ~35x. Mutations were identified using the BRESEQ pipeline [3]. To detect potential duplication events we used ssaha2 [37] with the paired-end information. This is a stringent analysis that maps reads only to their unique match (with less than 3 mismatches) on the reference genome. Sequence coverage along the genome was assessed with a 250 bp window and corrected for GC% composition by normalizing by the mean coverage of regions with the same GC%. We then looked for regions with high differences (>1.4) in coverage. Large deletions were identified based on the absence of coverage. For additional verification of mutations predicted by BRESEQ, we also used the software IGV (version 2.1) [38].

### Phenotypic characterization of evolved clones

#### Growth in single carbon sources

The same samples of clones from the populations which were tested in the competition assays were used to estimate the growth curves in different carbon sources. Two media were used: M9 Minimal Media (MM) supplemented with maltose 0.4% or with glucose 0.4%. The growth curve assays were performed on a Bioscreen C microplate reader, using a volume of 150μL per sample and an inoculum of ~10^4^ CFUs. Plates were incubated at 37°C with shaking before each optical density measurement (OD at 600nm). All growth measurements were repeated at least twice.

#### Fitness of effect of *fhuA* mutant under oxidative stress

To test if the mutation on the *fhuA* gene conferred some advantage to the evolved bacteria in specific selective pressures characteristic of the macrophage intracellular environment, we grew ancestral and mutant clones under oxidative and iron limitation stresses. We combined different concentrations of Fe^3+^ (Iron (III) Chloride hexahydrate, Alfa Aesar #A16231) with the ferrichrome siderophore (Ferrichrome Iron-free, Santa Cruz Biotechnology # sc-255174) and added hydrogen peroxide (H_2_O_2_) (Hydrogen Peroxide solution 30% (w/w), Sigma # H1009). Ferrichrome captures iron III and the resulting complex is imported into the cell by the FhuA outer membrane transporter. Excess iron inside the cell may be detrimental in the presence of H_2_O_2_, due to the Fenton reaction. In the KO mutant of fhuA, ferrichrome-dependent uptake of iron does not occur, which could provide an advantage to the bacteria when exposed to oxidative stress.

The mutant used for this experiment was the sequenced clone of population C (*fhuA* KO and *selC* IS), which was compared to an ancestral clone.

The two clones were first grown in M9 Minimal Media supplemented with 0.4% Glycerol in an orbital shaker at 37°C with 230rpm, to an OD_600nm_ of 1 (stationary phase). The cultures were then diluted and grown again in the same conditions until they reached an OD_600nm_ of 0.4–0.6 (exponential phase). After normalization to the same O.D, samples were diluted 100x, divided in equal parts and centrifuged at 3220 g for 30 minutes, before being re-suspended in the same growth media either supplemented, or not, with Fe^3+^ and Ferrichrome at two different concentrations: 0μM Fe^3+^ + 100μM Ferrichrome and 100μM Fe^3+^ + 1000μM Ferrichrome. Samples were acclimatized at 37°C with agitation for ~15 minutes before the addition of H_2_O_2_ to a final concentration of 2 mM. Samples were then left at 37°C without agitation and collected after 1h, washed in PBS 1X and plated on LB agar. Plates were incubated for 16h at 37°C, followed by CFU counting.

#### Analysis of *rfaI* conservation in other *E*. *coli* strains

A list of all sequenced strains of *E*. *coli* was retrieved from the European Bioinformatics Institute database (www.ebi.ac-uk/genomes, accessed on April 2014). The meta-information for all the strains (*i*.*e*., laboratory origin, pathogen or commensal) was manually curated by accessing several different public microbial databases. The fasta sequences were retrieved for each of the genes comprising the *rfa* locus in *Escherichia coli* BW2952 (*rfaBCDFGIJLPQSYZ* and *waaAU*) and then BLASTed against the sequenced genomes of the genus *Escherichia* and *Shigella* (74 genomes in total), using Biopython. If, for a given strain, the query was returned as empty, we considered the gene to be absent. Otherwise, the gene was present but with varying degree of conservation, although not below 82% similarity.

#### *In vivo* test for increased pathogenesis

C57/BL6 mice, aged 7–10 weeks (in house supplier, Instituto Gulbenkian de Ciência) were given food (RM3A(P); Special Diet Services, UK) and water *ad libitum*, and maintained with a 12 hour light cycle at 21°C. The animals were infected intra-peritoneally with 2x10^7^ CFUs of either the ancestral clone or evolved clone I (carrying two IS insertions in *fhuA* and *rfaI*) diluted in 100μl of PBS. Furthermore, as a control, in each experimental block we injected a group of 2–3 mice with 100 ml of saline (these animals did not display any signs of disease). Mice were followed for 4 days post infection and their weights and temperatures were monitored daily. The infections were performed in two blocks, with n = 3 mice per bacterial strain per block. A linear mixed effect model, with bacterial strain and day post-infection as factors and mouse as a random effect, was used to determine if significant increases in weight loss occurred in an infection with the evolved clone.

## Results and Discussion

### Dynamics of *E*. *coli* adaptation to intracellular life

We followed the evolutionary dynamics and adaptation of twenty independent populations of *E*. *coli* during repeated exposure to the intracellular environment of MΦs. The bacterial populations were all founded from an equal mix of two ancestral clones, which were isogenic except for a distinct neutral fluorescent marker. Under the hypothesis that periodic selection will dominate the pathoadaptive process, the occurrence and spread of a strong beneficial mutation in one of the clones with a given fluorescent marker will cause the extinction of all other clones and hence the loss of diversity at the marker locus [8,39]. A more complex pattern may emerge if adaptive mutations are very common and cause clonal interference [15], which may slow the loss of neutral variation [13], or if coexisting interdependent ecotypes emerge [16,40].

**Fig 1:**
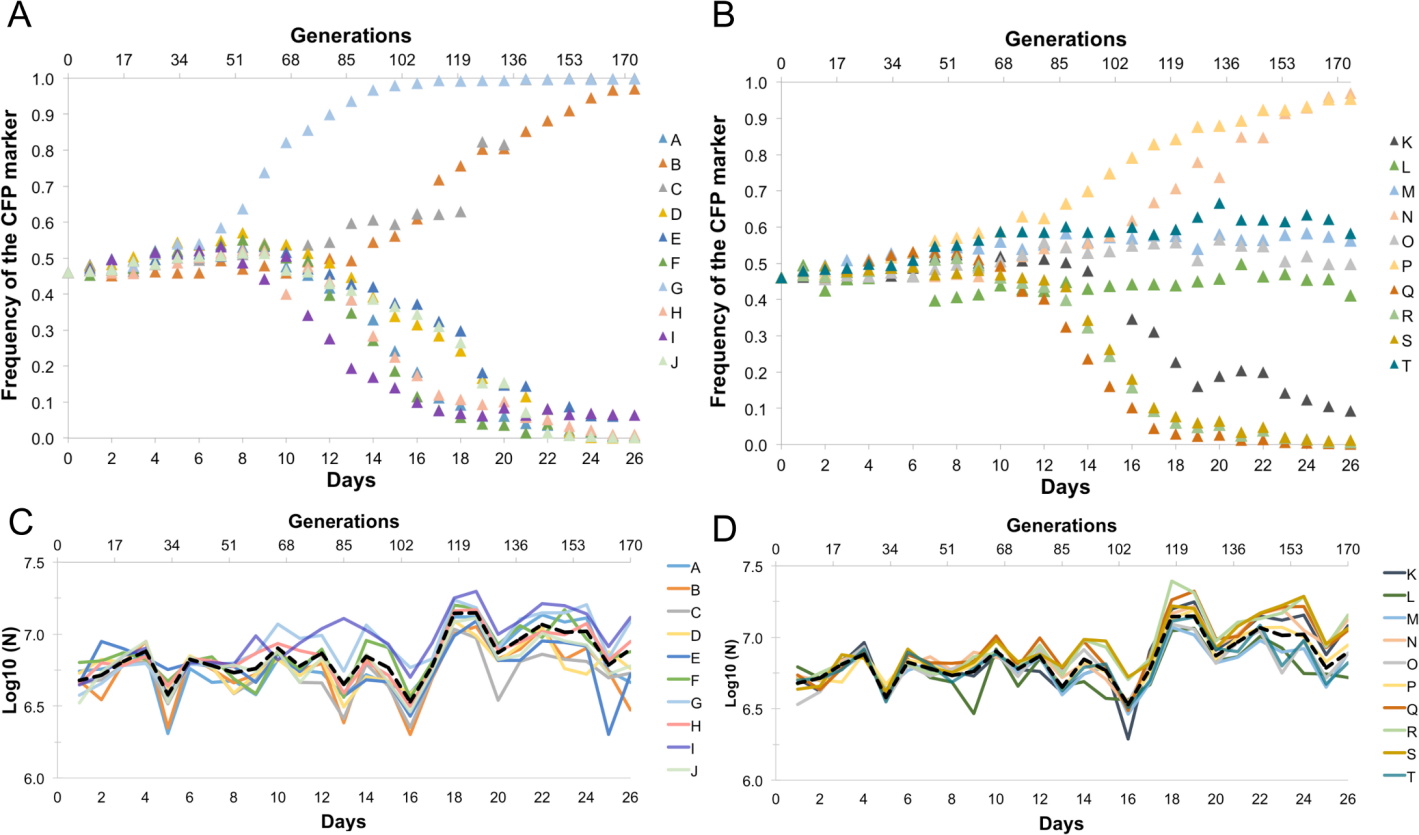
Evolutionary dynamics of the populations evolved within macrophages. Dynamics of frequency of neutral marker in the 20 replicate evolving lines (in (A) lines A to J; in (B) lines K to T) and variation in population size along the evolution experiment (in (C) lines A to J; in (D) lines K to T).

In our experimental evolution protocol, MΦs (10^5^/ml) were infected with *E*. *coli* for 24 hours, after which all extracellular bacteria were killed with gentamicin. 10^4^ bacteria sampled from the intracellular environment of MΦs were then used to infect new uninfected MΦs. The evolution experiment was followed for 26 days and the occurrence of adaptive mutations was detected through the observation of rapid and consistent changes in the frequency of the neutral marker (Fig 1A and 1B). After 10 days of propagation, consistent changes in frequency started to be detected in some populations and by day 15 most of the populations showed significant deviations from the initial marker frequency (15 out of 20 populations showed deviations above 10%), suggesting that beneficial mutations had spread through the populations (Fig 1A and 1B). During the 26 days of evolution, in only one of the populations (O) the deviation from the initial marker frequency was less than 10%. A significant increase in the total number of bacteria after infection was also detected after 100 generations in all the lines evolved (Fig 1C and 1D and S1 Table). The increase in carrying capacity (K) of the evolving populations tends to be observed in synchronicity with the changes in the marker frequency, indicating that this fitness trait is being modified by occurring adaptive mutations.

### Pathoadaptation occurs at a high rate and involves strong effect mutations

The rapid and consistent changes in the frequency of each of the fluorescent alleles imply the occurrence of strong beneficial mutations. Assuming a simple model of positive selection we can estimate their rate and effect through the deviations of the neutral markers [15,41]. We have estimated these key evolutionary parameters using two different approaches: Marker Divergence Analysis [8,15], which summarizes the neutral marker dynamics using two parameters: the effective mutation rate (U_e_) and the effective selection coefficient (S_e_), by fitting simulations to the marker dynamics. This method, which assumes all mutations generated within a replicate population to have a given fixed effect, has been shown to perform acceptably for scenarios of low clonal interference [14] and summarizes the adaptive dynamics of all the populations by a single value of U_e_ and S_e_. The second method, Optimist [41], determines the likelihood that the frequency of a neutral marker results from a given number of haplotypes, arising at a given time and segregating with a particular effect. For each particular replicate population, the number of haplotypes that best explains the marker frequency dynamics is chosen by the lowest Akaike Criteria, resulting in a distribution of the number of haplotypes, as well as their effects, for all the replicate populations.

From the dynamics in Fig 1A and 1B, the best estimates of U_e_ and S_e_ were 1.6x10^-6^ (mutations per genome per generation) and 0.26, respectively. Using the method implemented in Optimist, we find a mean increase in fitness of mutations of 0.09 (see Table 1 for the estimated parameters and S1 Fig for the corresponding simulated dynamics that best fit the experimental data). These estimates of the rate and strength of fitness effects of adaptive mutations can be compared with those obtained in bacterial adaptation to other environments, and using similar methods of inference. *E*. *coli* rates of adaptation to compensate for the costs of antibiotic resistance were found to lay in the range of 10^−7^, and mean *s* in the range of 5 to 15%, dependent on the strain that evolved [8,41]. Using a different experimental system with more neutral markers, [9] estimated higher rates of compensatory mutations to resistance U~10^−5^, with mean effects of 2.5 and 3.6% dependent on the resistance mutation. It is becoming well established that the distribution of effects of adaptive mutations markedly depends on the genetic background. For *E*. *coli* strains with the same genetic background as the ones used here, but adapting to a simpler environment (Luria-Bertani rich medium) Hegreness et al [15] found U_e_ = 2x10^-7^ and S_e_ = 0.05. These estimates are considerably smaller than the estimates found here, when the strain faces harsher conditions. Since the same strain and the same method of estimation were used in our experiment, the comparison of the combined estimates demonstrates that the evolutionary parameters strongly depend on the environment. They furthermore support the idea that in more stressful environments, where strong biotic interactions prevail, higher rates and effects of adaptive mutations are to be expected [42].

**Table 1:** Inferred selective effects of beneficial haplotypes.

Population	# of Mutations	W Mut#1	Time Mut#1	W Mut#2	Time Mut#2
**A**	**2**	0.101	28	***0*.*073***	***28***
**B**	**1**	***0*.*054***	***0***		
**C**	**1**	***0*.*109***	***63***		
**D**	**1**	0.063	14		
**E**	**1**	0.056	7		
**F**	**2**	***0*.*091***	***21***	0.134	35
**G**	**2**	***0*.*123***	***7***	0.099	14
**H**	**2**	0.134	42	***0*.*109***	***42***
**I**	**2**	0.090	7	***0*.*094***	***42***
**J**	**1**	0.071	28		
**K**	**2**	0.141	63	***0*.*121***	***63***
**L**	**0**				
**M**	**1**	***0*.*027***	***0***		
**N**	**1**	***0*.*053***	***0***		
**O**	**2**	***0*.*054***	***14***	0.063	35
**P**	**2**	***0*.*073***	***0***	0.060	7
**Q**	**2**	0.112	35	***0*.*111***	***77***
**R**	**2**	0.101	35	***0*.*103***	***77***
**S**	**2**	0.151	56	***0*.*124***	***63***
**T**	**2**	***0*.*043***	***7***	0.089	98

The number of mutations inferred for a specific population is indicated in the 2^nd^ column. W mut#1 and T mut#1 (3^rd^ and 4^th^ columns) indicate, respectively, the inferred fitness improvement and time of appearance (in generations) of the first mutant. W mut#2 and T mut#2 (5^th^ and 6^th^ columns) indicate the same inferred parameters for the second mutant. Cells with values in italic bold indicate a mutation inferred in the CFP background.

### Competitive fitness assays reveal two distinct strategies of pathoadaptation

**Fig 2:**
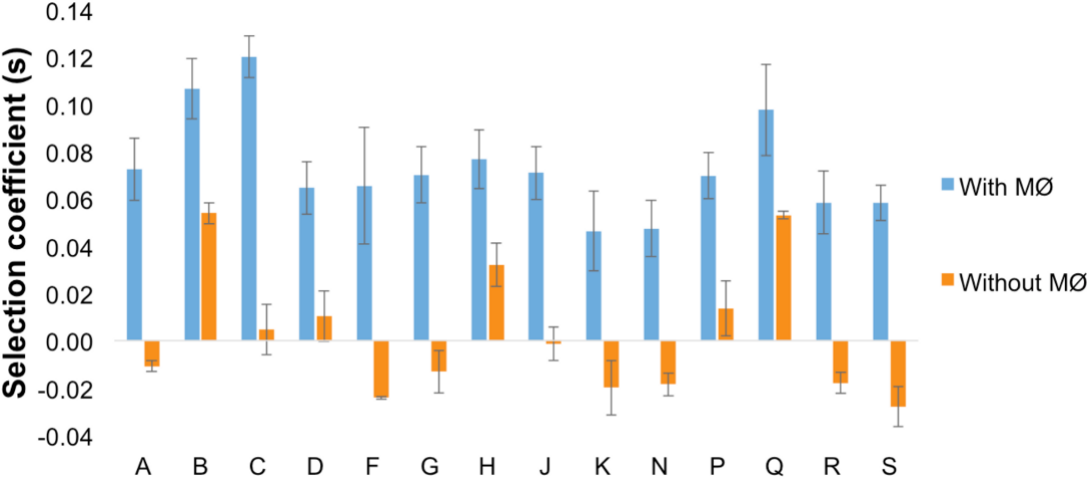
Fitness measures of evolved clones. In blue, the competitive fitness of evolved populations in the presence of MΦ: evolved clones (a sample of 30 from each indicated population) were competed against the ancestral clone (1:1). In orange, competitive fitness assay in the absence of MΦ. Error bars correspond to 2SE.

The changes in frequency of each fluorescent allele suggested a strong effect of the beneficial mutations that occurred. To support this inference and directly estimate the strength of these mutations, we performed competitive fitness assays, in the presence of macrophages, of evolved clones against the ancestral strain marked with a different fluorescence. Fig 2 shows that all populations exhibit a significant fitness increase and are therefore better adapted to the environment with macrophages. The mean competitive fitness increase observed was 7%, with a minimum of 5% and a maximum of 12% (Fig 2, blue bars). These values are in close agreement with those estimated from the changes in marker frequency alone and assuming the simplest model of positive selection (mean of 9% with a minimum of 4% and a maximum of 15% (see Table 1 and S2 Table). Although there is a slight overestimation of the fitness effects inferred by the marker deviations, they can be explained by a number of reasons. Firstly, and contrary to what is assumed by the model, selection in such complex environments might not be constant, leading to non-linear effects of beneficial mutations. Secondly, theoretical approaches are known to overestimate the effects of mutations when there is more than one mutation (i.e., cases of higher clonal interference) [14,41]. Finally, the AIC criteria (see methods) might be too stringent in selecting models that postulate an increased number of haplotypes, which will lead to stronger effect mutations. Nevertheless, both fitness measures are in agreement that the most likely form of selection taking place in this environment involves sweeps of beneficial mutations of strong effects.

One possible trait that could be expected to evolve as an adaptation to the selective pressure imposed in this experiment would be an increased ability to grow in the abiotic environment external to the macrophages (RPMI). If a variant with increased fitness in RPMI would emerge, then its frequency outside macrophages could increase and dominate the population; a likely scenario if such a mutant did not have any cost inside macrophages nor in the external environment as it becomes conditioned by those cells. To determine whether the evolved populations increased in fitness in RPMI, *i*.*e*. in the absence of macrophages, we performed competitive fitness assays against the ancestor in the medium alone (Fig 2, orange bars).The results show that in 3 out of 14 populations there is, indeed, a significant fitness increase in the abiotic environment, suggesting that increasing growth in RPMI can be beneficial in the presence of macrophages. We note that during the evolution the abiotic environment outside macrophages is likely to change, so a mutant which is beneficial in RPMI may change its advantage as this medium becomes conditioned by the presence of macrophages. The results also show a correlation between the changes in fitness in the absence of macrophages to the increase in fitness in their presence (Pearson r = 0.688, P = 0.0065, Fig 2). In half of the populations (A,F,G,K,N, R and S) a clear trade-off was detected (Fig 2). For these cases, accumulation of mutations with significant advantage in the presence of macrophages led also to a decreased competitive ability in their absence. This indicates a specialization in the transition to intracellular life. Together, the results suggest different adaptive strategies adopted by similar bacteria adapting independently to the same environment, but with distinct genetic mechanisms evolved to cope with the same antagonistic interaction.

### Genetic basis of the intracellular adaptation reveals common evolutionary paths

**Fig 3:**
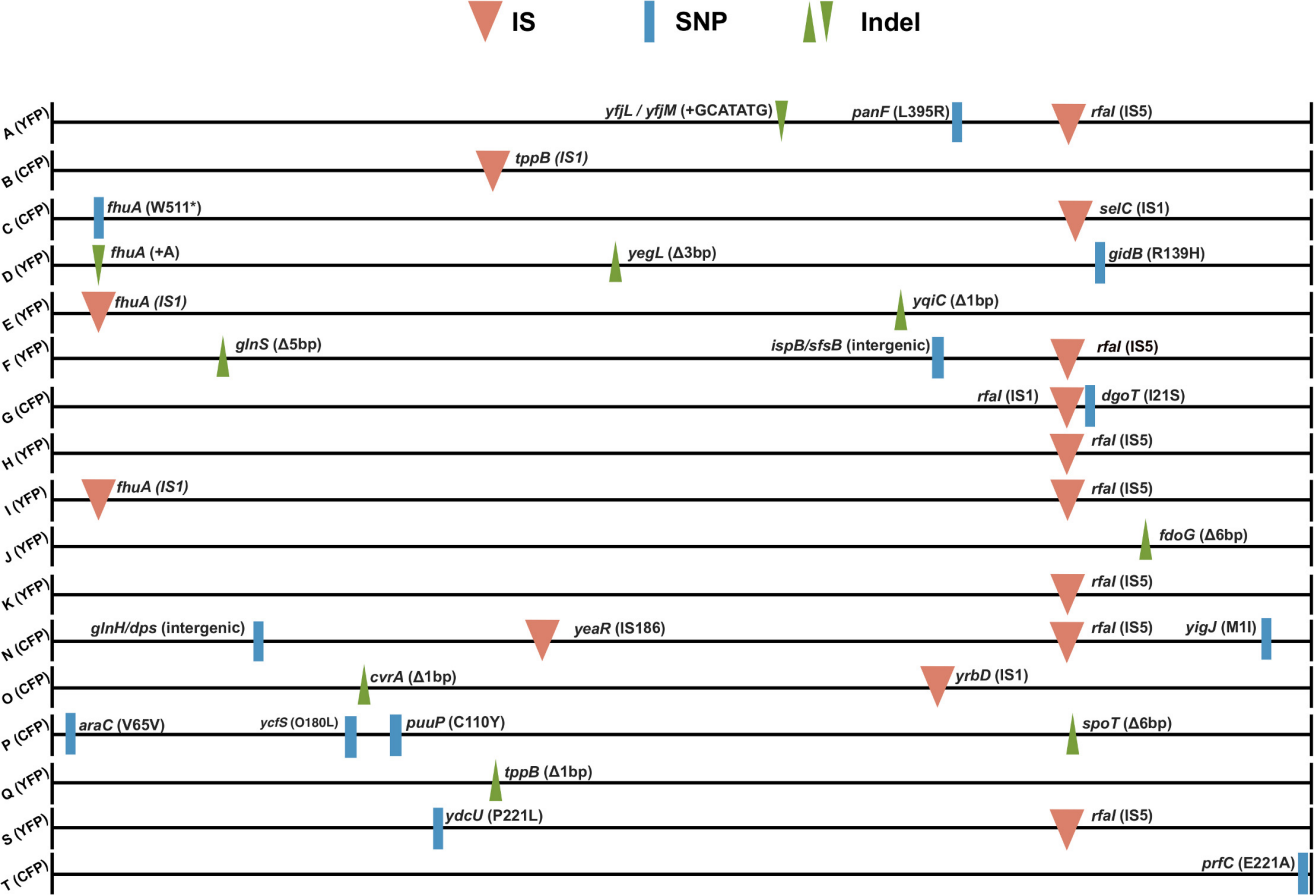
Genomic maps of the mutations detected in the sequenced evolved clones. Red inverted triangles represent insertions of IS elements, blue lines mark single nucleotide polymorphisms and green triangles denote small insertions (pointing downwards) or deletions (pointing upwards). All events have either the aminoacid changes associated (for SNPs), the IS element inserted or the small number of base pairs deleted or inserted.

Given the dynamics of neutral markers observed (see Fig 1A and 1B), the short duration of the experiment (~175 generations) and the estimates of only a few of beneficial mutations being responsible for the adaptive process (see Table 2), we predict that each population is dominated by a single clone with one or two mutations. In order to unravel the number of genetic changes that occurred and to reveal the underlying evolutionary paths taken by the populations, we performed whole genome sequencing of independently evolved clones. The evolved strains and their ancestor were sequenced to a minimum of 16x coverage on the Illumina Miseq platform. Table 2 shows the genetic changes detected and Fig 3 their position along the chromosome. Overall, 25 different mutational targets were detected amongst the adapted clones. As expected, each clone carries an average of 2 mutations. Most of the mutations occurred in coding regions and 14 out of 34 in total involved insertions of transposable elements IS1, IS5 and IS186. The first two have been found to transpose at higher rates than other elements [43] and are therefore more likely to contribute to adaptation. Among the gene targets for the mutations detected, two occurred in 4 and 8 clones (*fhuA* and *rfaI*, respectively) and one occurred in two independently evolved clones (*tppB*). Parallelism is a hallmark of adaptation since the probability that mutations in the same gene increase in frequency by random chance in at least two independent lines, over such a short period, is very low [44,45]. Given the parallelism observed involving the gene targets *rfaI* and *fhuA*, we can safely assume that these changes are adaptive. Furthermore, the change hitting the coding region of *tppB* (either through an insertion or by a small deletion) in two independent clones, together with it being the sole detected mutation (B CFP and Q YFP) suggests that loss-of-function of *tppB*, coding for a proton-dependent transporter of di- and tri-peptides could be an important pathoadaptive mutation.

**Table 2:** Mutations identified in the sequenced clones.

Clone (Coverage)	Genome Position	Gene	Mutation	Annotation	S	Tradeoff without MΦs
**A YFP (36x)**	2649245	*yfjL/yfjM*	+GCACTATG	intergenic (-258/+102 nt)	7%	Yes
	3293960	*panF*	T→G	L395R (CTG→CGG)		
	3689096	*rfaI*	IS5 + 4bp	coding (312/1020 nt)		
**B CFP (35x)**	1603229	*tppB*	IS1 + 5 bp	coding (378/1503 nt)	11%	No
**C CFP (73x)**	169014	*fhuA*	G→A	W511* (TGG→TAG)	12%	No
	3722624	*selC*	IS1 + 8bp	non coding (47/95 nt)		
**D YFP (20x)**	168462	*fhuA*	+A	coding (980/2244 nt)	6%	No
	2039547	*yegI*	Δ3 bp	coding (1944-1946/1947 nt)		
	3809621	*gidB*	C→T	R139H (CGC→CAC)		
**E YFP (93x)**	3070100	*yqiC*	Δ1 bp	coding (91/291 nt)		
	168929	*fhuA*	IS1 + 8bp	coding (1447/2244 nt)		
**F YFP (16x)**	3219882	*ispB/sfsB*	G→A	intergenic (131/-197 nt)	7%	Yes
	608471	*glnS*	Δ5 bp	coding (396-400/1665 nt)		
	3689096	*rfaI*	IS5 + 4bp	coding (312/1020 nt)		
**G CFP (40x)**	3758025	*dgoT*	A→C	I21S (ATC→AGC)	7%	Yes
	3689065	*rfaI*	IS1 + 8bp	coding (343/1020 nt)		
**H YFP (20x)**	2541599	*hscA*	Δ3 bp	coding	8%	No
	3689096	*rfaI*	IS5 + 4bp	coding (312/1020 nt)		
**I YFP (148x)**	167493	*fhuA*	IS1 + 8bp	coding (11/2244 nt)		
	3689096	*rfaI*	IS5 + 4bp	coding (312/1020 nt)		
**J YFP (346x)**	3971199	*fdoG*	Δ6 bp	coding (2311-2316/3051 nt)	7%	No
**K YFP (28x)**	3689096	*rfaI*	IS5 + 4bp	coding (312/1020 nt)	5%	Yes
**N CFP (34x)**	75049	*glnH/dps*	A→T	intergenic (-295/+109 nt)	5%	Yes
	4410884	*yjgJ*	G→A	M1I (ATG→ATA)		
	1769910	*yeaR*	IS186 + 10bp	coding (122/360 nt)		
	3688872	*rfaI*	IS5 + 3bp	coding (536/1020 nt)		
**O CFP (35x)**	1129729	*cvrA*	Δ1 bp	coding (296/1737 nt)		
	3223632	*yrbD*	IS1 + 11bp	coding (9/522 nt) / intergenic (-1/+4)		
**P CFP (29x)**	70580	*araC*	T→C	V65V (GTT→GTC)	7%	No
	1072984	*ycfS*	G→A	O180L (CCG→CTG)		
	1247732	*puuP*	C→T	C110Y (TGT→TAT)		
	3708996	*spoT*	Δ6 bp	coding (247-252/2115 nt)		
**Q YFP (34x)**	1602868	*tppB*	Δ1 bp	coding (17/1503 nt)	10%	No
**S YFP (29x)**	1404545	*ydcU*	C→T	P221L (CCG→CTG)	6%	Yes
	3689096	*rfaI*	IS5 + 4bp	coding (312/1020 nt)		
**T CFP (30x)**	4546582	*prfC*	A→C	E221A (GAA→GCA)		

Coverage for each clone is indicated in the first column. The 6^th^ column (S) indicates the selective effect of the evolved clones in the presence of macrophages, compared to the ancestor, and the 7^th^ column indicates whether there is a selective tradeoff (i.e., fitness in the absence of macrophages is lower than ancestor).

### Loss of *rfaI* leads to a strong selective sweep during adaptation to macrophages

**Fig 4:**
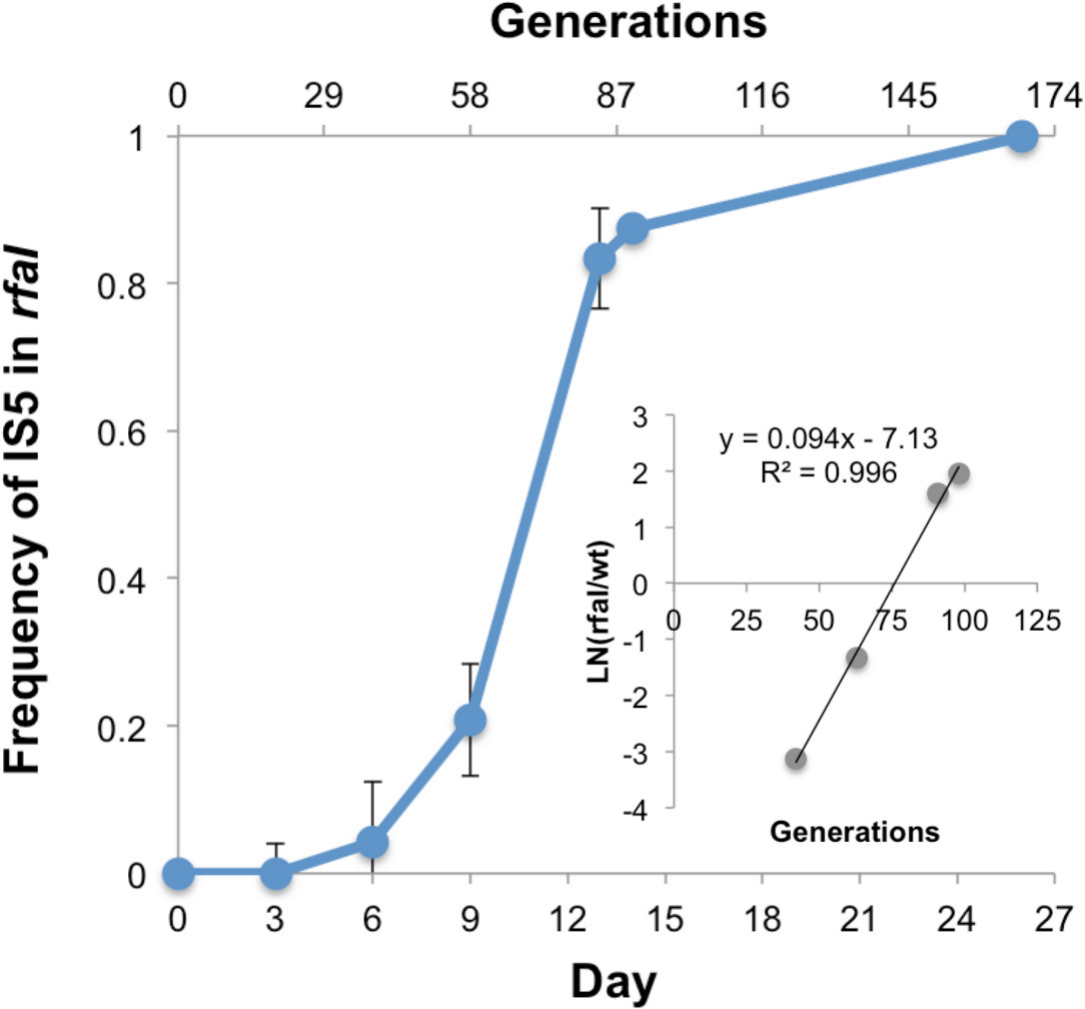
Invasion of an adaptive mutation at the *rfaI* locus. The blue line shows the selective sweep leading to the fixation of an IS5 insertion likely causing gene loss of function. In the inset an estimate of the selection coefficient (*s*) of this mutation is given. *s* is the slope of the logarithm of the ratio between the number of clones carrying the mutation and those with the wild-type allele.

In 47% of the evolved populations, mutations in *rfaI* (all of them IS insertions presumably leading to gene inactivation) were detected, suggesting this to be a preferential target and, therefore, one with high beneficial effect. We followed the emergence of this adaptive mutation in one of the adapted populations (population I), by targeted PCR for the presence of IS5 element in *rfaI*, as this element had been identified in the evolved clone sequenced from this population. Fig 4 shows that the mutation could be detected by day 6, with a frequency 4.1% (SE 0.04) and rapidly swept to fixation, being detected in all tested clones (n = 60) at day 26. We could directly estimate its selective effect, from its initial change in frequency, to be 0.09 (see inset of Fig 4).

We also found a strong correlation between the presence in a clone of a mutation in *rfaI* and a competitive tradeoff (i.e., benefit in the presence of macrophages, but disadvantage in their absence) of the populations where that clone mutation emerged (p<0.01, Pearson correlation). *rfaI* is a glycosyltransferase and part of the lipo-polysaccharide (LPS) synthesis machinery present in bacteria. LPS are unique and complex glycolipids that provide characteristic components in the outer membranes of bacteria and as such are a critical component of their interaction with cells from the immune system [46]. The *rfa* locus itself is composed of 15 different genes, which are responsible for generating different parts of the LPS structure [47]. *rfaI* is involved in the outer part of the core oligosaccharide, connecting the lipid A (inner part) and the O-antigen (outer part) of LPS. Since the strain used in this study is devoid of O-antigen, the outer part of the core is the LPS terminal section, and it is likely acting as one of the main interfaces between the bacterial cell and the cells from the immune system. Modifications in the LPS structure, and the outer core in particular, are known to modify the behavior of bacterial cells regarding adhesion to epithelial cells and biofilm formation in enterohemorrhagic *E*. *coli* [48,49], and intracellular invasion of different serovars of *Salmonella enterica* [50]. Moreover, mutations in the outer core structure of *Brucella abortus* can induce pro-inflammatory responses and enhanced macrophage activation [51]. Interestingly, many genes in the *rfa* locus itself are a target for bacterial persistence in *E*. *coli* [52], and the operon seems to be poorly conserved in a vast group of *E*. *coli* pathovars, with several pathogenic (and non-pathogenic) strains missing many of its genes (including *rfaI*) (see Analysis of rfaI conservation in other E. coli strains (section) in Material and Methods). This suggests that, in these strains, the *rfa* genes could be a common mutational target. Together, both our results and these observations seem to indicate an important role of the LPS structure both in the interaction with the immune system and in the transition to a pathogenic lifestyle, implying the changes in *rfaI* detected in 8 independently evolving populations as a recurrent pathoadaptive target.

### *fhuA* pathoadaptive mutant is beneficial under the combined pressure of iron limitation and oxidative stress

**Fig 5:**
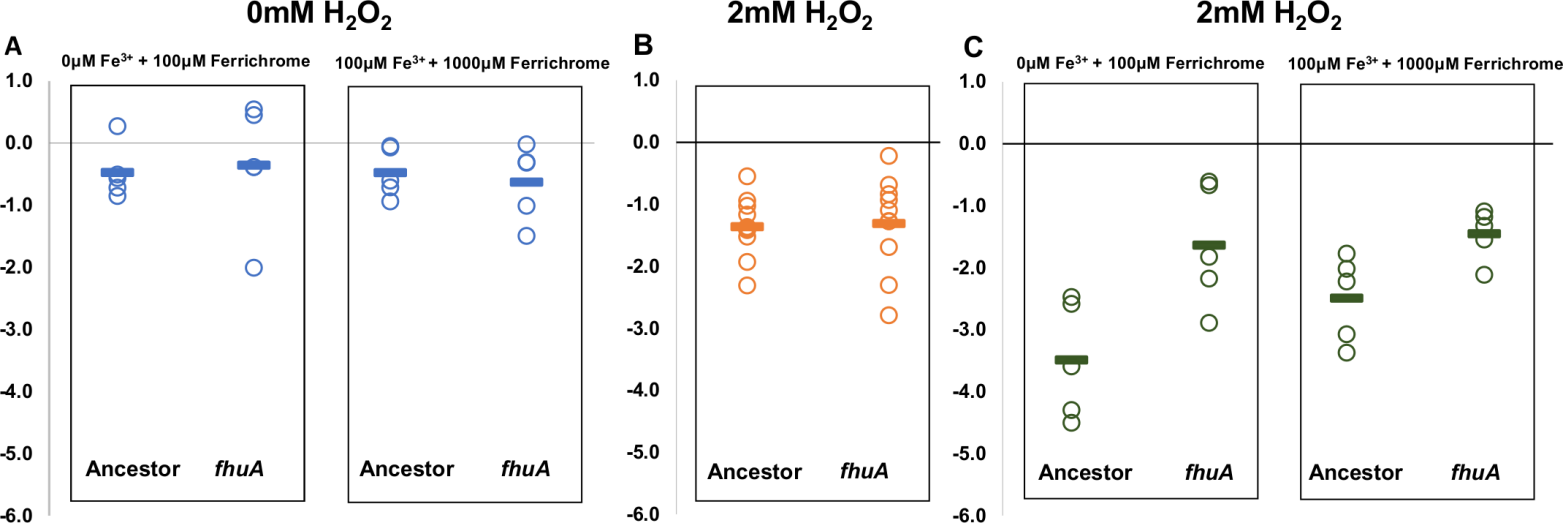
Bacterial survival under iron and or oxidative stress. Log_10_(Number of bacteria after 1h)- Log_10_ (Number bacteria at 0h) in the environments: (A) Effect of Fe^3+^ depletion tested in minimal media supplemented with the indicated concentrations of Fe^3+^ and Ferrichrome; no significant difference between evolved and ancestral clones could be detected (T-test, P = 0.8 and P = 0.6, n = 5) (B) Effect of H_2_O_2_ stress tested in minimal media supplemented 2mM of H_2_O_2_; no significant difference between evolved and ancestral clones (T-test, P = 0.8, n = 10). (C) Fitness advantage of evolved clone is detected under both selective pressures, i.e. minimal media supplemented with the Fe^3+^, Ferrichrome and 2mM of H_2_O_2_ (T-test, P = 0.016, P = 0.02, n = 5)

The beta barrel protein FhuA is involved in the active transport of ferric siderophores across the outer membrane of Gram-negative bacteria [53]. Iron homeostasis is crucial to the lives of both bacteria and macrophages therefore both cells have exquisite mechanisms to achieve physiological levels of iron and to keep it in a safe intracellular non-toxic form. Although oxidative stress can be generated by aerobic respiration, it is also one of the microbicidal pressures generated by the macrophages in the harsh phagosomal environment. Superoxide and hydrogen peroxide (O_2_^-^ and H_2_O_2_) are moderately reactive oxygen species, however, upon interaction with iron, the highly reactive hydroxyl radical (OH^-^) can be created (Fenton reaction) (reviewed in [54]). Since phagocytosed bacteria can face high levels of oxidative stress inside macrophages we tested the survival of a *fhuA* mutant in different conditions regarding the presence/absence of H_2_O_2_ (2mM H_2_O_2_) and different concentrations of Ferrichrome plus Fe^3+^. Ferrichrome is a siderophore which binds iron III and enables it to be transported through the FhuA outer membrane transporter. We find that in the presence of Ferrichrome alone (100μM) or complexed with Fe^3+^ (100μM Fe^3+^, 1000μM Ferrichrome), survival of the *fhuA* mutant is indistinguishable from that of ancestral bacteria (Fig 5A). A similar result was obtained in the presence of H_2_O_2_ (Fig 5B). A fitness advantage of the evolved clone was however detected in an environment comprising oxidative stress in conjunction with Ferrichrome, or with Ferrichrome and Fe^3+^. Under these conditions the survival of *fhuA* mutant clones is significantly increased in relation to that of ancestral bacteria (P = 0.001 and P = 0.008 respectively) (Fig 5C). The difference between the mutant and the ancestor is observed even in the absence of Fe^3+^ supplementation. This could be justified by the fact that bacteria are able to grow under limited amounts of this element, which is present under most biological conditions [55].

These results therefore indicate that this mutation may have evolved to decrease the amount of OH^-^ inside the bacterial cells.

### An *in vitro* evolved double mutant of *rfaI* and *fhuA* shows increased pathogenic potential *in vivo*

We tested for increased virulence of one of the MΦ adapted clones. This clone carries two of the mutations that repeatedly emerged during the evolution: an insertion into *rfaI* and an insertion into *fhuA* (clone I Fig 3). By infecting mice in the intra-peritoneal cavity with either the ancestral strain or the double mutant we find that, although mouse survival is similar for both strains, the weight loss caused by the infection of the evolved strain was significantly higher (P = 0.046 for strain and P = 0.003 for time, in a linear mixed effects model, with mouse as a random effect and strain and day of infection as factors, see S2 Fig). Given that the increased pathogenic potential of the double mutant was significant but not very strong we did not test each of the single mutants. Besides weight loss, a common phenotype to assay pathogenicity *in vivo*, we also measured temperature, but found no significant difference.

### Pathoadaptation to macrophages can lead to metabolic trade-offs

**Fig 6:**
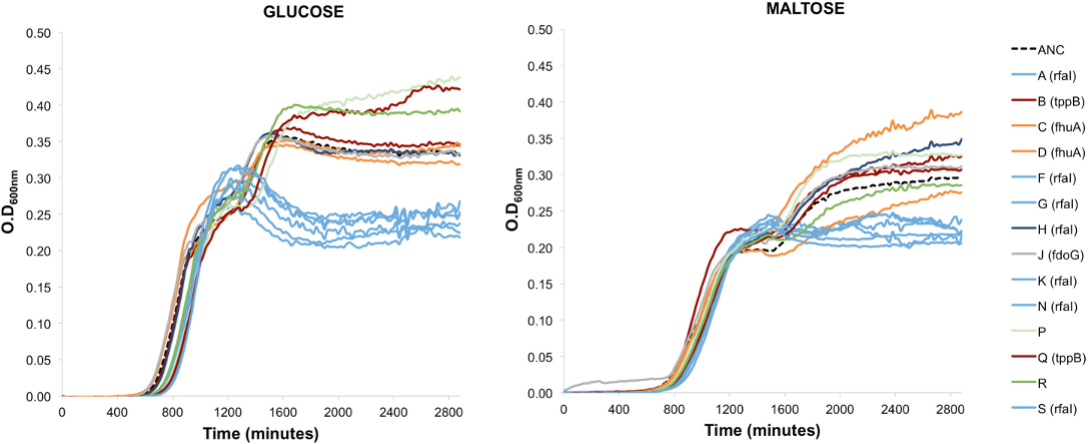
Growth curves of evolved populations and the ancestral strain in minimal media with maltose or glucose (0.4%). Each color on the growth curves represents similar patterns amongst the populations.

Bacteria fully adapted to intracellular life tend to have small genomes [56]. Amongst the species of *E*. *coli*, *Shigella* strains have undergone a considerable amount of genome reduction [57]. During its evolution from an extracellular inhabitant of the mammalian gut to an intracellular pathogen, *Shigella* accumulated a plethora of pseudogenes, with genes coding for carbon utilization, cell motility, transporter or membrane proteins more likely to become inactivated [58]. While part of this gene loss may be the outcome of intensified genetic drift and inefficient selection, it can also be the result of positive selection for loss of anti-virulent functions, constituting adaptive losses in the intracellular niche [56,59]. Such losses may entail antagonistic effects in extracellular environments. We tested the adapted clones for differences in their ability to grow on single carbon sources and found that some exhibited a strong metabolic trade-off when growing on either glucose or maltose (Fig 6). We found that all the clones carrying the pathoadaptive loss of *rfaI* failed to reach high carrying capacity on minimal media with either of the sugars. In contrast, the mutants with pathoadaptive mutations in *tppB*, involved in the transport of peptides, showed increased growth in maltose (Fig 6, red lines). Interestingly, subsequent mutations on the *rfaI* mutant background restore the ability to grow to similar levels as the ancestor, showing that the pleotropic effects of such pathoadaptation can be compensated to better grow on both poor and rich media.

## Conclusions

Characterizing the evolutionary and genetic mechanisms underlying the transition from commensal to pathogenic lifestyle is paramount in understanding the particularities of what makes pathogens dangerous and often fatal to their hosts. Here, we followed the evolution of a commensal strain of *E*. *coli* under the selective pressure imposed by the intracellular niche of MΦs to identify the most probable paths of this adaptation. All evolved populations show an increased ability to survive in the presence of macrophages, as the result of acquisition of strong beneficial mutations, which we estimate and measure to be around 7 to 10%, on average. The characterization of their genetic basis unveiled mutation that were highly likely to be pathoadaptive mutations, namely those involving changes in LPS, crucial in the interaction with the immune system, and in iron metabolism, essential for both protecting against high levels of toxicity and to acquire the necessary resources to survive. Given the strong pressure imposed in our experimental system, our results show that commensal bacteria are able to acquire adaptations to increase their intracellular survival at a fast pace. Importantly, the adaptive mutations identified in this study suggest possible new therapeutic targets to counteract pathogenic intracellular parasites.

## Supporting Information

S1 Fig Inferred Evolutionary Dynamics. Simulated dynamics of the model of positive selection [41] with the parameters that provide the best fit to the data of changes in marker frequencies (displayed as points). Each color represents an independently evolved population.
(PDF)

S2 Fig *rfaI* and *fhuA* double mutants increase weight loss in mice. The change in weight of mice (as a percentage) after intra-peritoneal infection with ancestral or evolved (clone I) bacteria.
(PDF)

S1 Table Increase in bacterial loads along the experiment. The majority of lines show a significant increase in bacterial loads. The slope of Log_10_(CFUs) along the 26 days of evolution, from a linear regression, is indicated in the 1^st^ column and P value of slope indicated in the 2^nd^ column.
(XLSX)

S2 Table Correspondence between experimental and inferred fitness. The experimental values measured through competitive fitness assays are indicated with their errors (2SE), along with the fitness inferred through the marker dynamics of the respective population. In the majority of populations (with the exceptions of populations B, K and S), the two measures are either in agreement or the inferred fitness is slightly overestimated (see the main text for discussion).
(PDF)
