# Supplementary figures and images for: Trade-Offs of Escherichia coli Adaptation to an Intracellular Lifestyle in Macrophages
Source: PLoS One. 2016 Jan 11;11(1):e0146123. doi: 10.1371/journal.pone.0146123 (PMC4709186; doi:10.1371/journal.pone.0146123)

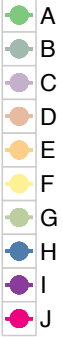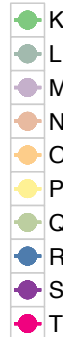

Supplement: S1 Fig — Simulated dynamics of the model of positive selection [41] with the parameters that provide the best fit to the data of changes in marker frequencies (displayed as points). Each color represents an independently evolved population. (PDF) [file pone.0146123.s001.pdf]

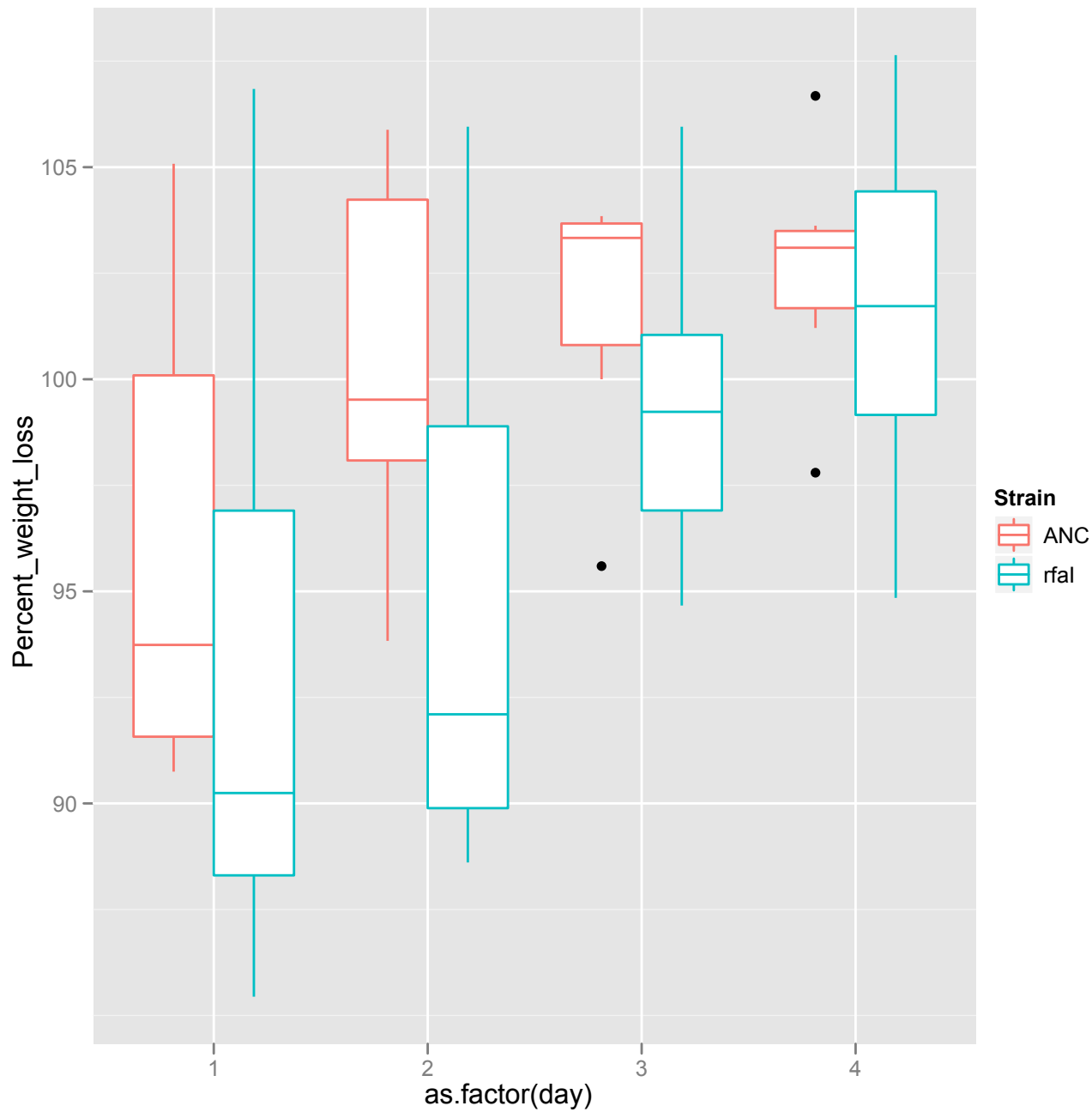

Supplement: S2 Fig — The change in weight of mice (as a percentage) after intra-peritoneal infection with ancestral or evolved (clone I) bacteria. (PDF) [file pone.0146123.s002.pdf]
