# Supplementary material for: Trade-Offs of Escherichia coli Adaptation to an Intracellular Lifestyle in Macrophages
Source: PLoS One. 2016 Jan 11;11(1):e0146123. doi: 10.1371/journal.pone.0146123 (PMC4709186; doi:10.1371/journal.pone.0146123)
Supplement: S2 Table — The experimental values measured through competitive fitness assays are indicated with their errors (2SE), along with the fitness inferred through the marker dynamics of the respective population. In the majority of populations (with the exceptions of populations B, K and S), the two measures are either in agreement or the inferred fitness is slightly overestimated (see the main text for discussion). (PDF) [file pone.0146123.s004.pdf]

	Experimental	Inferred
A YFP	[0.06 - 0.08]	0.1
B CFP	[0.10 - 0.12]	0.05
C CFP	[0.11 - 0.13]	0.11
D YFP	[0.05 -0.07]	0.06
F YFP	[0.05 - 0.09]	0.13
G CFP	[0.06 - 0.08]	0.12
H YFP	[0.07 - 0.09]	0.13
J YFP	[0.06 - 0.08]	0.07
K YFP	[0.03 - 0.07]	0.14
N CFP	[0.04 - 0.06]	0.04
Q YFP	[0.8 - 0.12]	0.11
R YFP	[0.05 - 0.07]	0.1
S YFP	[0.05 - 0.07]	0.15
